# The lived experience of clozapine discontinuation in patients and carers following suspected clozapine-induced neutropenia

**DOI:** 10.1186/s12888-023-04902-w

**Published:** 2023-06-08

**Authors:** Ebenezer Oloyede, Danielle Dunnett, David Taylor, Ivana Clark, James H MacCabe, Eromona Whiskey, Juliana Onwumere

**Affiliations:** 1 Pharmacy Department, South London and Maudsley NHS Foundation Trust, London, UK; 2 Department of Psychosis Studies, Institute of Psychiatry, Psychology & Neuroscience, King’s College London, King’s College London, London, UK; 3 Institute of Pharmaceutical Science, King’s College, London 5th Floor, Franklin-Wilkins Building 150 Stamford Street, London, SE1 9NH UK; 4 National Psychosis Service, South London and Maudsley NHS Foundation Trust, London, UK; 5 NIHR Biomedical Research Centre for Mental Health South London and Maudsley NHS, London, UK; 6 Department of Psychology, Institute of Psychiatry, Psychology and Neuroscience, King’s College London, London, UK; 7 University of Oxford, Department of Psychiatry , Warneford, United Kingdom

**Keywords:** Clozapine, Discontinuation, Neutropenia

## Abstract

**Background:**

Clozapine is the treatment of choice in refractory psychosis. In most countries, clozapine must be stopped indefinitely if white blood cells fall below a defined threshold during routine monitoring. Despite evidence of severe adverse consequences of clozapine discontinuation, published accounts on the lived experiences and perspectives of patients and carers are scarce.

**Method:**

We completed semi-structured interviews with patients (n = 4) and family carers (n = 4) on experiences of clozapine cessation following suspected drug-induced neutropenia. Interviews were audio-recorded, transcribed and analysed thematically.

**Results:**

The two overarching themes comprised:(i) stress of clozapine below threshold neutrophil results and (ii) patient and carer priorities.

**Conclusions:**

There is a suggested need for evidence-based pharmacological and psychological approaches to support patients and carers after clozapine cessation. Such approaches will minimise the potentially negative physical and emotional sequela in the aftermath of a below threshold neutrophil result and reduce the likelihood of experiencing additional health and social inequalities after clozapine discontinuation.

**Supplementary Information:**

The online version contains supplementary material available at 10.1186/s12888-023-04902-w.

## Introduction

Clozapine is the most effective antipsychotic for treatment-refractory psychosis [1]. In many countries around the world, patients prescribed clozapine are required to undergo haematological monitoring indefinitely [2]. Haematological monitoring is intended to mitigate against the rare but potentially fatal risk of clozapine-induced agranulocytosis (CIA) (prevalence ~ 0.4%) [3]. The nature of clozapine monitoring systems, including absolute neutrophil count (ANC) and/or white cell counts (WCC) thresholds for categorising readings vary widely between countries [2]. As an example, in the US, absolute neutrophil count (ANC) thresholds for clozapine discontinuation are ≥ 1.0 × 10^9^/L (or ≥ 0.5 × 10^9^/L if there was a history of benign ethnic neutropenia [BEN]). Whereas thresholds are 0.5 × 10^9^/L higher in the United Kingdom and countries regulated by the European Medicines Agency. Notably, the overwhelming evidence indicates that US monitoring thresholds do not compromise patient safety [1, 4–6]. In the event of a below threshold haematological reading, patients are required to immediately stop clozapine treatment [1]. Treatment may then be stopped indefinitely or restarted depending on the follow-up blood test results [1]. Approximately 1 in 10 patients discontinue clozapine due to suspected clozapine-induced neutropenia (CIN) [7].

The discontinuation of clozapine can have several negative clinical consequences, including withdrawal symptoms, deterioration in mental state, and reduced antipsychotic response [8, 9]. The sudden and unplanned nature of treatment discontinuation after suspected CIN may potentially have greater implications for both patients and carers. Accordingly, public health authorities have prioritised identifying ‘true’ CIN, as the current system is believed to vastly overestimate CIN and underestimate non-clozapine causes such as benign neutropenia [1, 4, 10, 11].

At present, the cumbersome nature of investigations for these potential differentials coupled with clinician hesitancy means that safely restarting treatment (i.e. clozapine rechallenge) is often delayed, if done at all [4]. To date, there has been little qualitative exploration of the lived experience, including reported needs, from patients and family carers following a below threshold haematological result and having to stop clozapine [12]. Understanding these experiences may assist care providers to provide optimal support and resources for patients and key others in their networks. Therefore, in people with lived experience of treatment resistant psychosis, their family and carers, we aimed to qualitatively explore their perspectives of discontinuing clozapine treatment after a haematological abnormality.

## Methods

### Study setting and ethical considerations

This was a qualitative, cross-sectional study which used semi-structured interviews. The study was conducted in the South London and Maudsley NHS Foundation Trust (SLaM), a large Inner London hospital, and approved by the local Drugs and Therapeutic Committee (approval number: DTC/2022/35). SLaM provides mental health services to 1·2 million people across four South London boroughs.

### Design

A reflexive thematic analysis methodology [13] was used for this study, to allow for the identification of themes across the dataset that could give specific ideas for systemic change [14].

### Interview

An interview topic guide was developed and used for all interviews by one author (EO), which was informed by clinical expertise, wider reference to the literature [8, 9] and iteratively refined via ongoing discussions with the research team. All participants were encouraged to introduce issues of importance not covered by the topic guide (see supplementary material for further details). Data generation and analysis were conducted in parallel using constant comparative technique with incoming data informing following interviews [15]. Data collection continued until the point of information power, which was classified regarding how well the data collected addressed the aims of this study, rather than when no new data emerged [16, 17].

### Participants

Patients and carers of those who previously experienced a red below threshold neutrophil result while treated with clozapine between January to September 2022 were recruited for interviews. This 6 month time span was chosen to include individuals who may have been rechallenged on clozapine after a confirmed red below threshold result with sufficient time to recall their experiences [1]. Participants were initially identified by EO and EW from the clozapine registry. Once permission to approach the patient was secured, the patient was approached by EO to obtain informed written consent. Sociodemographic and clinical information (e.g., age, sex) was retrieved from medical notes. Interviews were held via Microsoft teams. Participants were compensated £10 for their time.

### Data Analysis

The interviews were analysed using Reflexive Thematic Analysis [13] within a critical realist framework, which views meaning and experiences as subjective and influenced by social and cultural context. An inductive approach was used to analyse the data. Thus, analysis was conducted in a bottom-up approach, allowing the data to lead the formulation of themes, rather than predefining themes or utilising a theoretical approach [18].

Participants were aware of the researcher’s (EO) status when taking part in the interviews but were informed that it was their own experiences that were of relevance to the research. Participants were also reassured that all views given were valid and important, to prevent them from feeling they had to provide only positive views. There were no existing relationships between the research team and participants. To ensure that this influence was acknowledged during the research process, the lead author employed reflective practice in the form of continuous debriefing with the wider research team (JO, E.W, DD).

Interviews were audio-recorded and transcribed verbatim. Identifying information was removed at transcription and recordings were destroyed. The two analysing authors (DD, JO) were guided by the explicit content of the data rather than making assumptions or looking at existing concepts [13, 14], as this approach felt it would be most informative for systematic change.

The six phases of thematic analysis outlined by Braun and Clarke were followed [18]. The analysing authors (DD, JO) achieved data familiarisation by reading and re-reading transcripts several times, followed by manual coding and final clustering and synthesis of codes to form themes.

Coding drew out key themes, words, and phrases and themes were generated independently prior to analysing authors meeting to discuss findings. No software was used during the analysis. It comprised constant comparison, with the relationships between codes explored alongside an analysis of each code across the transcripts. Conducting the analysis concurrent with data collection ensured iterative interaction between data and analysis to enhance reliability [19].

Through regular meetings, with time between for reflection, themes were revisited and reviewed. Quality checks were performed by comparing a random sample of recordings to transcripts. The wider research team met to review and refine the themes, which were discussed against the coded data and the final themes and subthemes were agreed. A final consensus was reached on the themes that best reflected the data.

Overall reliability was established by probing the relationship between each individual transcript and the themes across the interviews as well as through discussions within the research team to forge shared interpretations. Drawing upon multiple researcher perspectives is advocated as a way of increasing the credibility and therefore the trustworthiness, of the final analysis [15, 20].

## Results

### Participants

From the twelve patients who had a recorded neutropenic event during the study period, four (1 male and 3 females) agreed to participate (33%). Individual interviews were conducted with four patients and four related carers. The mean length of interviews was 43 min.

Patients had an average age of 39 years and were prescribed clozapine for a mean of 9 years (range: 2–18). All patients had a diagnosis of paranoid schizophrenia. One of the patients had been re-challenged on clozapine after the haematological event. One patient discontinued clozapine treatment after the haematological event due to frequent monitoring requirements.

### Themes

Analysis of interview data identified two core themes that speak to (1) the negative psychological sequelae of recording haematological aberrations with clozapine treatment and (2) calls for improvements to care and service delivery. (Table 1): report these.

**Table 1:** The overall theme and subthemes that emerged from the interview

Theme	Subtheme	Participants’ quotes
Psychological stress of clozapine below threshold neutrophil results	• Traumatic experience	[patient 1] “It is a very traumatic experience and I just have to grin and bear it…I think it is the word red (below threshold neutrophil result) alert the worry I will have to come off clozapine again.”” [carer 1] “She had three red ((below threshold neutrophil) results and during these times it has been extremely traumatic.” [carer 2] “We were brought to our knees; it was a dreadful time” [carer 4] “Every time, he is taking off his clozapine he has got to go straight back to square one again, you know, and its harrowing, its harrowing for him because then he has got people around his house every night giving him his medication and putting it up by 25, to get it back to where it should be on his clozapine, you know, it is just harrowing for him he gets so fed up with it” [Carer 4] “Yeah, it affects us we are worried, you know, we don’t want him going back into somewhere else because he is doing so well on his own and he is doing his cooking, his cleaning and he has his own place, everything he wanted he has got so far, you know”
	• Immediate discontinuation	[patient 1] “I was really upset and worried because I did not want to go into hospital and on both occasions [of receiving a red ((below threshold neutrophil) result], I went into hospital, I did not want to be an inpatient… I was really upset and worried as I did not want to go into hospital, but off clozapine I just couldn’t handle life.” [carer 3] “There were catastrophic consequences, within 72 hours the whole period of recovery was completely, totally reversed, she had a complete relapse.” [carer 1] “Each red result she had to immediately come off the medication, literally stopped there and then, resulting in physical and mental illness, within a day, she was violently sick and very unwell mentally…By the second day, she was completely hostile and paranoid, and thought Jesus was on an island somewhere paying her benefits, I mean really ill, she was trying to cope, and she was hardly sleeping.”
	• Proactive response	[carer 1] “The most important thing is immediate action, we were told on a Friday night, so we were just abandoned. We didn’t hear from him [consultant] until Wednesday, the next week or something. It’s crazy, just shocking. In hindsight, I would have not stopped clozapine, I would have said no, we are not going to stop it until you put a plan in place…A drastic decision was made to cut her off clozapine, there was no plan in place if she relapses, what medication is available instead of clozapine and medication to help her sleep, there was no plan there for six days.”
Patients’, families’ and carers’ priorities	• Staying in the community	[patient 2] “I really wanted to be treated in the community, I think the community needs to improve medication services so that they can treat patients with a red (below threshold neutrophil) alert in the community, instead of going into hospital…I do not mind being a patient in the community, but I just do not like being in hospital.” [patient 4] “I know when I come off clozapine, I need to be assessed but what I would love to happen is the medication team need to develop so they can treat a patient with a red (below threshold neutrophil) result in the community.” [carer 2] “It can just rewind everything [recovery/progress], putting people back in hospital, which is the last thing anyone would want.” [carer 4] “ Yeah, he has his own place and when they’ve taken him off clozapine they want him back in hospital but he is happy he has his own independence and you know, he can go down the shops and buy his own food and do his own cooking and his own washing and stuff like that and that is all beneficial but if you take that away and you put him into [hospital site] and that’s such an awful thing, you know, but he is always being monitored then he hasn’t got much of a life has he as it is, you know, because of his mental state but yeah that’s some of a life where he can do his own thing”
	• Contingency plans for red result	[carer 1] “You must immediately put an immediate action plan into place, and I don’t mean in a week or two, I mean at the same time, you cannot remove the clozapine without putting in support, I think that is crucial.” [patient 3] “I just want everybody to be educated about me, about the protocol (G-CSF)” [carer 2] “I think the frequency of blood tests should be dramatically increased before removing clozapine, for a specified period and see if those alarming results remain stable.” [carer 4] There has always been a communication problem ever since mum and dad passed away its been nothing but headaches for me and my brother [brothers name], he has had team meetings with doctors and staff and yet like I said the doctor says something and then that’s it, that’s as far as it goes, it doesn’t go to A then to B and from B to C, it stays with A and I just find it so ridiculous.
	• Information on outcomes after discontinuing clozapine	[patient 3] “I didn’t have anybody to talk to, I feel that even in the community they do not explain anything about clozapine.” [patient 1] “They need to tell me what will happen if I get a red result, whether I have to have blood tests or to start again.” [carer 1] “I do not remember having any information on what to expect when she came of it” [patient 3] “It would be helpful, with what withdrawal symptoms you may have, understanding if somebody comes off clozapine how it is going to affect them physically”. [carer 4] “Yeah, but we didn’t receive any information about it whatsoever from day one, like I said my mum use to do it, she use to go with him for the blood tests and so she would know what was going on, you know, but because we can’t make it down there, it’s like I said we just don’t get any information whatsoever from his care coordinator or social worker, she doesn’t receive anything from anyone. We don’t receive any information from the clozapine clinic whatsoever” [carer 2] “I mean was completely off; was high, completely high we are sitting in a funeral and emotions are all over the place, you know, and like I said as far as we knew was still on these tablets, but hadn’t been on the tablets since before Christmas day” [carer 4] “You know, when you’re sitting there, and she is an emotional state, we all are emotional states but especially and when we got to the wake, we started to realise that he has not been on his tablets, you know, he was taken off them before Christmas, but like I said there was no feedback to let us know, so we could keep a better eye on him and stuff. I was absolutely furious about it…”
	• Monitoring for adverse effects	[carer 1] “The problem we had with it was that nobody did anything at all and haven’t done in six months to determine the low count, nothing whatsoever…Lack of evidence and overwhelming data to suggest that low count had nothing to do with clozapine, but we were given the red ((below threshold neutrophil) results and were simple told on a Friday night we had to stop clozapine.” [carer 2] “Nobody monitors or monitored her for 18months now. If those life threatening, potentially life-threatening side effects have no importance and have no reason for anybody to ask a single question about, why is it that red (below threshold neutrophil) results are taken so dramatically differently, it’s a huge contradiction.” [carer 4] “Then those time they take him of his clozapine and not all the time is it down to the clozapine itself its down to infections like an ear infection.”

## Discussion

### Study findings

The eight participants in our study described physical and psychological challenges from recording neutropenia and its aftercare. Key themes included the negative psychological impact of suspected CIN and patient and care priorities. Despite potentially life-changing consequences, patients and carers described a lack of effective communication and care planning after receiving a haematological aberration with clozapine. The preliminary findings from our modest pilot investigation suggests patients and carers may experience cessation of clozapine owing to neutropenia as a catastrophic event.

### Comparison to other studies

To date, studies of patient, family or carer experiences with clozapine treatment have primarily focussed on clinical aspects such as symptom changes, tolerability and mandatory haematological monitoring and have often neglected the psychological impact of recording neutropenia, sudden unplanned treatment discontinuation and its aftercare [12]. Notwithstanding, studies in other medical specialities support our observation that an illness’s diagnostic uncertainty and potential recurrence may result in trauma-related psychological disturbances [21]. For example, fear of cancer recurrence is a significantly distressing problem that affects a substantial number of patients with and survivors of cancer and is often the most frequently endorsed unmet need [21]. Within this perspective it is plausible that comparable clinical needs are shared with individuals and families who experience suspected CIN, particularly once rechallenged on treatment.

### Themes

For patients and carers, recorded haematological aberrations with clozapine treatment were associated with feelings of worry, uncertainty, anxiety, and service abandonment. For carers, specifically, the sense of being neglected by services and being left in the dark about what was likely to occur in their relative’s health and mitigation plans and/or solutions were particularly highlighted. Patients and carers described experiencing elevated levels of distress and adjustment difficulties and described the process that followed the neutropenia notification and their experiences as being traumatic. Patients and carers felt unsupported and not adequately informed about the potential consequences of recording neutropenia with clozapine treatment. Patients and carers expressed a lack of contingency and prevention planning, despite the potential prognostic impact of clozapine discontinuation and the role that information was likely to have in reducing levels of anxiety and uncertainty. Our analysis suggests that clozapine monitoring systems distinctly prioritise physical health over mental health and deemphasise long-term mental health care for patients with TRP. Previous studies have demonstrated how haematological thresholds and unrecognition of BEN can lead to unnecessary clozapine discontinuation. Of note, BEN is believed to predominantly associated with people of African, middle eastern, and Caribbean ancestry, and so the problem of inappropriate clozapine cessation may disproportionately affect people of these ancestries [10, 11].

It is crucial to identify and address the unique needs of families and carers during a haematological aberration with clozapine. For example, our interviews revealed a period of heightened anxiety during follow-up investigations and monitoring. Understandably, the realisation that a relative may experience a potentially fatal reaction coupled with the need to abruptly stop the only treatment that has succeeded in symptom alleviation can be disabling and associated with varied emotional reactions including anxiety, worry and fear. Notably, carers described disappointment with healthcare providers’ responses to their lived experiences and expressed concerns and found their lack of understanding, avoidance and poor communication particularly difficult to accept. Uncertainty with care plans, processing medical information, and complex decision-making regarding whether to rechallenge clozapine made the initial period of aftercare particularly overwhelming. Recognising and addressing these needs should be equally prioritised by care providers and remain consistent with treatment and good practice guidelines.

### Limitations

Our study is limited by the small sample size and its confinement to one care setting. Nevertheless, to our knowledge, this is the first study of its kind to explore patient and carer experiences of a haematological aberration with clozapine. Moreover, CIA is a rare event (incidence 0.4%) [22]. It is of note that our patient interviewees were community dwelling and engaged with their pharmacological treatments, including clozapine, and most had a favourable view of clozapine before the haematological aberration. It is unclear if individuals with unfavourable views of clozapine and/or residing in different settings (e.g. psychiatric inpatient care) would express share similar views. It is conceivable, however, that irrespective of thoughts about clozapine, the diagnostic follow-up and the impact of abrupt clozapine discontinuation (e.g. withdrawal symptoms), are consistently burdensome among all patients. Furthermore, our study is limited by our involvement in data collection as healthcare professionals.

### Clinical implications and future studies

Our findings have important implications for clinical practice. Around 1 in 10 people taking clozapine are forced to stop taking it because of haematological toxicity [7]. However, it is estimated that 1 in 250 people experience a true agranulocytosis [22]. The vast majority of those stopping clozapine can be safely re-exposed to clozapine, indicating that clozapine had been stopped unnecessarily. The catastrophic nature of sudden cessation of clozapine should be considered when stopping clozapine [9]. Equally, providing psychosocial support for carers and patients is crucial. Thoughtful and careful assessment and the development of appropriate treatment pathways can optimise the detection and management of distress and traumatic stress. For example, clozapine discontinuation after a suspected haematological aberration should not be automatic. Instead, this should prompt clinicians to investigate differential causes of neutropenia such as viral infections, concomitant medication, or BEN. Moreover, service providers should strongly consider providing clinicians with access to training and resources such as evidence-based guidelines and expert opinion to support patient care, including identification and treatment of potential withdrawal symptoms and alternative treatment options during clozapine interruption. Second, interviewees highlighted the importance of being able to remain in the community, receiving a contingency plan, and timely information on potential outcomes after clozapine discontinuation. It seems important to consider these issues and anticipate the potential occurrence of haematological aberrations with clozapine. Patients and carers should be counselled on haematological monitoring requirements and potential consequences prior to treatment initiation. Interestingly, categorising test results using a traffic-light system was considered unnecessarily anxiety-provoking, especially considering potential non-clozapine causes. Universal use of US classification may address this issue [1]. Future studies should investigate the impact of the recurrence of such events on patient and family perspectives and the impact of professional support.

## Electronic supplementary material

Below is the link to the electronic supplementary material.

Supplementary Material 1

## Data Availability

The data that support the findings of this study cannot be made publicly available for confidentiality reasons. Data are however available from the corresponding author upon reasonable request.
