# Supplementary material for: The lived experience of clozapine discontinuation in patients and carers following suspected clozapine-induced neutropenia
Source: BMC Psychiatry. 2023 Jun 8;23:413. doi: 10.1186/s12888-023-04902-w (PMC10249299; doi:10.1186/s12888-023-04902-w)
Supplement: Supplementary file 1 — Supplementary Material 1 [file 12888_2023_4902_MOESM1_ESM.docx]

**Living in the red zone: A focus group study exploring patient’s experience with haematological aberrations during clozapine treatment**

| **QUESTIONS** | **Prompts** |
| --- | --- |
| - **Clozapine treatment involve having regular blood tests** | |
| 1. What is your understanding about the role of blood tests in clozapine treatment? | What information have you been given about blood tests with clozapine |
| 1. What has it been like for you having clozapine blood tests? | Has there been anything you have found particularly helpful/less helpful?  Or  difficult/less difficult?  Or  Positive / negative |
| - **Clozapine treatment requires regular blood tests because clozapine can cause a drop in the number of your white blood cells.** - **White blood cells are used by the body to fight infections.** - **If you don’t have many white cells, your body will find it hard to fight infections such as ‘flu or a sore throat.** | |
| - **The clozapine system uses a ‘traffic light’ coding to rank the results from your blood tests. The system uses a ‘red’, ‘amber’ and a ‘green’ light system.** - **Red means your white blood cells count is much lower than normal. If this happens, clozapine must be stopped straight away.** - **Amber means your white blood cell count is a little lower than normal. If this happens, you will need a blood test twice a week until it goes back to normal (‘green’) or goes ‘red’.** - **Green means your white cell count is normal and you can carry on taking clozapine.** | |
| 1. What are your thoughts about the traffic light system? |  |
| 1. When you hear the term neutropenia or agranulocytosis, what does it mean to you? |  |
| 1. What have been your experiences of having a red result? | What was it like hearing about it?  How did you feel about having to stop clozapine?  Did you think stopping clozapine may impact your health or relationships with others?  Did you experience any withdrawal effects when stopping clozapine?  How did you feel about having to have daily blood tests? |
| 1. How did you feel when…. 2. What particular thoughts ran through your mind when…. 3. Following the news of ….., were there anything that you stopped doing or started doing? | In your opinion, if you think about your own experience or imagine someone else, what might you find helpful from staff member or service if you were to have a red result? |
| 1. How would you describe your relationship with the health professionals and services that were involved in monitoring and stopping clozapine? | Did you feel supported? What was the information given to you at that moment? Did you feel like you understood the information provided? |
| 1. What would have helped them or would help others in the future to come to a better collaboration between the professionals working in mental health care and the people with a red result? | What types of information might you find helpful?  How might you like to receive that information (e.g. particular format, particular person, plan for the future) |
| **CLOSING QUESTIONS** | |
| 1. What was the most important issue that we have talked about today? |  |
| 1. How have you found the process of talking today? Is there anything else you would like to add |  |
